# Which providers can bridge the health literacy gap in lifestyle risk factor modification education: a systematic review and narrative synthesis

**DOI:** 10.1186/1471-2296-13-44

**Published:** 2012-05-28

**Authors:** Sarah Dennis, Anna Williams, Jane Taggart, Anthony Newall, Elizabeth Denney-Wilson, Nicholas Zwar, Tim Shortus, Mark F Harris

**Affiliations:** 1 Centre for Primary Health Care and Equity, School of Public Health and Community Medicine, University of New South Wales, Sydney, NSW, 2052, Australia; 2 School of Public Health and Community Medicine, University of New South Wales, Sydney, NSW, 2052, Australia

**Keywords:** Health literacy, Lifestyle risk factor modification, Primary health care

## Abstract

**Background:**

People with low health literacy may not have the capacity to self-manage their health and prevent the development of chronic disease through lifestyle risk factor modification. The aim of this narrative synthesis is to determine the effectiveness of primary healthcare providers in developing health literacy of patients to make SNAPW (smoking, nutrition, alcohol, physical activity and weight) lifestyle changes.

**Methods:**

Studies were identified by searching Medline, Embase, Cochrane Library, CINAHL, Joanna Briggs Institute, Psychinfo, Web of Science, Scopus, APAIS, Australian Medical Index, Community of Science and Google Scholar from 1 January 1985 to 30 April 2009. Health literacy and related concepts are poorly indexed in the databases so a list of text words were developed and tested for use. Hand searches were also conducted of four key journals. Studies published in English and included males and females aged 18 years and over with at least one SNAPW risk factor for the development of a chronic disease. The interventions had to be implemented within primary health care, with an aim to influence the health literacy of patients to make SNAPW lifestyle changes. The studies had to report an outcome measure associated with health literacy (knowledge, skills, attitudes, self efficacy, stages of change, motivation and patient activation) and SNAPW risk factor.

The definition of health literacy in terms of functional, communicative and critical health literacy provided the guiding framework for the review.

**Results:**

52 papers were included that described interventions to address health literacy and lifestyle risk factor modification provided by different health professionals. Most of the studies (71%, 37/52) demonstrated an improvement in health literacy, in particular interventions of a moderate to high intensity.

Non medical health care providers were effective in improving health literacy. However this was confounded by intensity of intervention. Provider barriers impacted on their relationship with patients.

**Conclusion:**

Capacity to provide interventions of sufficient intensity is an important condition for effective health literacy support for lifestyle change. This has implications for workforce development and the organisation of primary health care.

## Background

The Australian National Primary Health Care Strategy [1] and Council of Australian Governments (COAG) Australian Better Health Initiative (ABHI) include key priority areas that focus on improved chronic disease prevention and screening of those with at least one risk factor for chronic disease [1]. Integral to achieving this is to increase health literacy particularly in relation to modifying the behavioural risk factors of smoking, nutrition, alcohol, physical activity and weight (SNAPW). The SNAPW risk factors are major contributing factors to the development of chronic disease worldwide [2-4] and are the focus of a number of international policy initiatives such as the US Healthy People 2010 initiative.

Health literacy is described as the cognitive and social skills which determine the motivation and ability of individuals to gain access to, understand and use information in ways which promote and maintain good health [5]. Low levels of literacy in the Australian population are a significant problem with recent figures indicating that less than half (48%) of the adult population reached the minimum level of literacy and numeracy required to function on a daily basis in today’s society [6-8]. This is consistent with research from UK and USA where 46% and 47% of the population respectively achieved the minimum level of literacy necessary [9,10]. The picture is even worse in people from low socioeconomic backgrounds and this further compounds their disadvantage [6,11]. Health literacy, as defined by Nutbeam [12] is likely to be present at much lower levels than literacy and numeracy. High levels of health literacy are associated with specific health promoting behaviours such as eating five portions of fruit and vegetables per day or being a non-smoker independently of age, education, gender, ethnicity or income [13,14].

Simply providing people with information alone about modifying SNAPW risk factors is not usually enough to bring about lifestyle change [15]. Rather, a partnership approach between patients and providers, based on shared decision making and good communication, may be necessary for developing a sense of confidence and ability to change [12,16]. Without adequate health literacy people may not have the capacity to self-manage their health and prevent the development of chronic disease through lifestyle risk factor modification.

In response to the National Primary Health Care Strategy [1] and National Preventative Health Strategy [17] there is a drive to improve the health literacy of Australians. Primary care is ideally placed to support lifestyle risk factor management and health literacy as 86% of the Australian population visit their GP at least once per year [18]. However addressing health literacy and SNAPW risk factor management in general practice is difficult; the average consultation time with a GP is 7–8 min shorter than the time necessary to provide smoking cessation counselling [19]. The tyranny of the urgent means that people may only present to the GP when sick leaving little or no time for prevention [20-22].

The developing role of practice nurses and allied health professionals in the prevention of chronic disease provides an opportunity to tackle SNAPW risk factor management and poor health literacy in those at risk of developing chronic disease. We know from a previous systematic review on skill mix that substituting GPs with health professionals such as nurses or pharmacists can be effective in disease management and health promotion in older people [23,24]. However it is not clear what impact the type of provider, such as dietician, diabetes educator or GP may have on the development of health literacy and associated SNAPW risk factor modification.

The aim of this systematic review and narrative synthesis is to determine how effective primary healthcare providers are at improving the health literacy of patients to make SNAPW lifestyle changes. A second aim is to discuss the drivers and barriers for health professionals trying to improving health literacy and risk factor modification in primary care.

## Methods

A systematic review was undertaken. Studies were identified by searching Medline, Embase, Cochrane Library, CINAHL, Joanna Briggs Institute, Psychinfo, Web of Science, Scopus, APAIS, Australian Medical Index, Community of Science and Google Scholar from 1 January 1985 to 30 June 2009. Health literacy and related concepts were found to be poorly indexed in many of the databases so a list of key words and text words were developed and retested for use in the different databases, terms used in the Medline search are listed in Table 1. Hand searches were also conducted of four key journals: Patient Education and Counselling, Health Education and Behaviour, American Journal of Preventive Medicine and Preventive Medicine. Systematic reviews identified in the process were read and all papers that met the inclusion criteria for this review were added to the list of papers. The bibliographies of experimental papers included were screened to identify additional studies.

**Table 1:** Terms used in Medline search

**Search Fields**	**Database specific terms (Text& MESH)**
**Health Literacy**	**Patient Education as Topic/ or exp Health Education/ or health literacy.mp. or exp Health Knowledge, Attitudes, Practice/ exp Patient Compliance/ exp Educational Status/(functional adj health adj literacy).tw.interactive health literacy.tw.critical health literacy.tw.**
**Outcomes**	**wrat.tw. realm.tw. tofhla.tw. hals.tw.social support scale.tw. diabetes care profile.tw. newest vital sign.tw. exp Physician-Patient Relations/ exp Self Efficacy/ exp rating scale/ or exp scoring system/ exp questionnaire/exp Psychological Rating Scale/**
**Primary Health Care**	**Primary Health Care/ exp Comprehensive Health Care/exp Patient Care Management/exp Family Practice/exp Physicians, Family/exp Community Health Services/(primary adj1 (care or health)).tw.(family adj1 (doct$ or medic$ or pract$ or physic$)).tw. (general adj1 pract$).tw. (gp or gps).tw.**
**Interventions**	**exp Health Promotion/ exp Motivation/motivation$ interviewing.tw. exp Behavior Therapy/ exp Risk Reduction Behavior/ exp Consumer Health Information/ exp Smoking Cessation/ self management.mp. exercise.mp. or exp Exercise/ brief intervention.mp.exp nutrition assessment/ exp Patient Education as Topic/ exp Self Care/ed [Education] exp Self Care/“group education”.mp. exp Education/**
**Lifestyle risk factors**	**exp Smoking/ec, pc [Economics, Prevention & Control] exp drinking behavior/ or exp alcohol drinking/ or exp feeding behavior/ or exp habits/ or exp health behavior/ exp Exercise/ exp Overweight/ exp Obesity/ exp risk factors/ exp Life Style/ exp Health Behavior/**
**Economic**	

There were several key definitions used to scope and focus the review.

1. Health literacy, represents basic skills (reading, writing and numeracy) which is functional health literacy. Interactive health literacy is the cognitive and social skills to actively participate in everyday living to extract information and derive meaning from different forms of communication, and to apply new information to changing circumstances to exert greater control over life events and situations (critical health literacy) [12].

2. Lifestyle risk factors for inclusion were: smoking, nutrition, alcohol, physical activity, and weight.

3. Primary health care was defined as first level care provided by a suitably trained workforce supported by integrated referral systems and in a way that gives priority to those most at need, maximises community and individual self-reliance and participation and involves collaboration with other sectors. It includes: health promotion, illness prevention, care of the sick, advocacy, and community development.

4. Providers were included in the review if they worked within a primary health care setting including general practice (family practice, primary care), community health, home nursing, private or public allied health, Aboriginal and multi-cultural health and health education and information.

5. A driver or barrier influences behaviour of a provider, organization or patient with regards to the uptake or use of an intervention. Two levels of drivers were defined [25]:

a. Primary drivers or barriers are system components which will contribute to moving the primary outcome.

b. Secondary drivers or barriers are elements of the associated primary driver. They can be used to create projects or change packages that will affect the primary driver.

Studies were included in the review if they were published in English, between 1985 and June 2009, included males and females aged 18 years and over with at least one SNAPW risk factor for the development of a chronic disease. The interventions had to be implemented within primary health care as defined and the studies had to report an outcome measure associated with health literacy (knowledge, skills, attitudes, self efficacy, stages of change, motivation and patient activation) and a measure of SNAPW behaviour change. We could not identify established tools for measuring interactive and critical health literacy so we looked to the self management literature for instruments that measure the concepts of self-efficacy, patient motivation, confidence and broader social support such as the Diabetes Self Efficacy Scale, the Social Support Survey and measures of Prochaska and DiClemente’s Stages of Change Model [26].

Intervention studies were included in the review if they were randomised, quasi randomised controlled trials, controlled before and after studies or interrupted time series. In addition non-experimental studies were included in an extraction of barriers and facilitators of health literacy and SNAPW risk factor management, see Table 2 for organisational framework for the review.

**Table 2:** Organisational framework for the review

					**Outcomes**		
**Patient characteristics**	**Intervention**	**Provider**	**Drivers / Barriers**	**Health Literacy**	Health Literacy Outcome^*^	**Behaviour Change**	**Cost Outcome**
Age	Information – written, video, oral, pictures,	Doctor	**Individual**	**Functional health literacy**	Disease knowledge,	**S**moking status	Intervention costs, Economic evaluation
Gender	Web based	Nurse	Language	Health related knowledge	Health related skills,	**N**utrition	
Ethnicity	Group self-management support, goal setting or education	Allied health	Knowledge/beliefs	Understanding	Health literacy score: TOFHLA, REALM, HALS, NVS	**A**lcohol use	
Socio Economic Status	Individual Motivational interviewing or Coaching		Ideologies	**Interactive health literacy**	Change in:	**P**hysical activity	
Education level	Telephone based (eg coaching)	Educator	Experiences	Motivation	Readiness to change	**W**eight	
Cognitive ability		Lay health worker	Medical conditions	Behavioural intentions	Attitudes		
Cultural factors		Multi-disciplinary team	**Social/community**	Empowerment	Knowledge		
Medical conditions			Environment	**Critical health literacy**	Patient activation		
Lifestyle risks			Social support	Cognitive skills	Measures of self-efficacy		
			Social norms	Social skills	Self management score		
			Networks	Personal skills			
			Culture/Traditions	Self-efficacy			
			Health system/provider				
			**Accessibility**				
			Interpreters				
			Incentives				
			Continuity of care				
			Time / workload				
			Communication skills				
			**Training**				
			Providers no.				
			Provider types				
			Up-to-date verbal and written information				
			Inter-sectoral				

The papers were screened by two researchers (AW and JT). A 10% sample of excluded studies was reviewed by a third reviewer (MH). Verification and data extraction were performed by two researchers (AW and JT), a quality assessment was performed using a published checklist [27] ( Additional file 1 and Additional file 2) by one reviewer (SD) and a 20% overlapping sample by a second researcher (AW). Data were extracted (AW and JT) into an MS Access™ database and included variables such as type of health professional, intervention description, duration and frequency of intervention and outcomes of interest. Interventions were coded into categories (group education, motivational interviewing and counselling, written material, mixed intervention, telephone or computer) and the intensity scored using a combination of frequency and duration of intervention. High intensity interventions were those with at least eight hours or contacts, medium intensity interventions had more than three hours or contacts but less than eight and low intensity interventions were those with up to three hours or contacts.

A vote counting approach to the synthesis was used. Each of the outcome measures of interest such as change in a SNAPW behaviour or health literacy measures were coded as significantly improved or not significantly improved based on the results reported in the paper for each outcome of interest. The outcomes were coded as a statistically significant improvement if the paper reported a positive change with a p ≤ 0.05. The tables report the total number of studies reporting that outcome measure as the denominator and the numerator is the total number of studies with a significant improvement in that outcome measure. This approach to the analysis has been used in other systematic reviews of complex interventions [23,28,29]

Drivers and barriers for providers involved in developing SNAPW health literacy were extracted from the 42 descriptive papers identified during the search by one researcher (SD) and the findings coded using the definitions from the Institute for Healthcare Improvement [25] and synthesised by two researchers (MH and SD). This review was conducted as part of a larger policy relevant review [30] and funded by a Stream 13 grant from the Australian Primary Health Care Research Institute.

## Results

**Figure 1:**
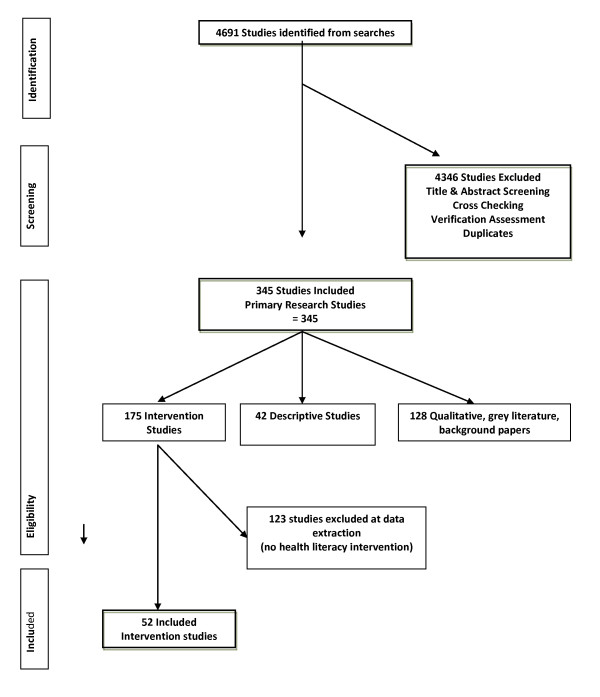
Review flow chart.

The database searches yielded 4691 papers that were assessed for inclusion in the review and after the screening and verification stages data were extracted from 52 papers that described intervention studies to address health literacy and lifestyle risk factor modification provided by different health professionals, see Figure 1 for PRISMA [31] flow chart. The characteristics of the included studies are in Table 3. In addition to the 52 intervention studies qualitative data on drivers and barriers were extracted from the 42 papers identified describing descriptive studies of health literacy and SNAPW risk factor modification, including facilitators and barriers.

**Table 3:** Characteristics of included studies

**Intervention Studies reporting**	**N**	**%**		**N**	**%**
**HL & SNAPW findings (N = 52)**					
**Publication Year**			**Setting**		
1988–1999	20	38.5	Community (General)	20	38.5
2000–2009	32	61.5	General Practice / Primary Care/ Community Health	28	53.8
Total	100	100	Other (hospital, not PHC)	4	7.7
**Countries**			**Health professionals**		
USA	30	57.7	Multi-disciplinary team	11	21.2
UK	7	13.5	Doctors	9	17.3
Australian/New Zealand	4	7.7	Other (not specified)	7	13.5
Sweden/Switzerland/Netherlands	9	17.3	Educator	6	11.5
Canada	1	1.9	Lay worker	6	11.5
Japan	1	1.9	Nurses	5	9.6
			Dieticians	1	1.9
			Computer	7	13.5
**Total**	**52**	**100**	**Total**	**52**	**100**

Most of the studies (71%, 37/52) demonstrated an improvement in health literacy, see Table 4. Overall, health literacy and SNAPW risk factor were both improved for 61% (14/23) of interventions to address nutrition, 54% (15/28) for physical activity, 43% (3/7) for weight and 40% (6/15) for smoking. When interventions were grouped according to the health professional providing the intervention, 33% (3/9) of the studies reporting interventions provided by doctors resulted in an improvement in health literacy compared to interventions provided by other health professionals such as dieticians, educators or nurses (92% 11/12) and multidisciplinary teams (91% 10/11). When the interventions were categorised into low, medium and high intensity it became clear that different types of health professionals tended to provide interventions of varying intensities according to our definition. For example, 71% (5/7) of the interventions provided by doctors were categorised as low intensity. These interventions tended to be motivational interviewing and counselling around smoking cessation and physical activity prescription and were often only one session with goal setting and were described as brief interventions [19,32-35]. In contrast 80% (8/10) of the interventions provided by nurses, dieticians or educators and 90% (9/10) provided by multidisciplinary teams were categorised as medium or high intensity. These interventions were often motivational counselling or group education programs that took place over a number of weeks and targeted smoking, nutrition or physical activity [36-52]. These interventions improved health literacy (10/11) although the effect on SNAPW risk factors was a little less with 8/11 reporting an improvement. Of the studies involving a lay worker, alone or as part of a multi-disciplinary team, 71% (5/7) targeted people from ethnic minority backgrounds. Overall, the included studies were of medium quality (36/52), 11 were high quality and five studies were of low quality. See Table 5 for a summary of the included studies.

**Table 4:** Studies by provider and type of intervention and outcome for SNAPW and health literacy

**Provider (No. of studies)**	**Intervention type (No. of studies)**	**Intervention Intensity**				**SNAPW outcomes (No. studies sig outcome/no studies measure SNAPW)**					**Health Literacy (No. studies sig outcome/No. studies measure HL)**
**Doctor (9)**		**H**	**M**	**L**	**NR**	**S**	**N**	**A**	**P**	**W**	
	Motivational interviewing or counselling (5)			3	2	1/1	0	0	2/4	0	3/5
	Mixed intervention (3)		2			0	1/1	0	0/1	0	0/2
	Written material (2)			2		1/1	1/1	0	0	0	0/2
	**Total**		**2**	**5**	**2**	**2/2**	**2/2**	**0**	**2/5**	**0**	**3/9**
**Nurse (5) Dietician (1) or Educator (6)**	Group education (4)	3	1			0/1	1/1	0	1/1	0	4/4
	Motivational interviewing or counselling (5)	2	1	2		1/2	0	0	4/4	0/1	4/5
	Mixed intervention (2)	1		1		0	1/1	0	0/1	0/1	2/2
	Telephone (1)	1				0	0	0	1/1	0	1/1
	**Total**	**7**	**2**	**3**		**1/3**	**2/2**	**0**	**6/7**	**0/2**	**11/12**
**Computer (7)**											
	Computer (2)			2		0/1	2/2	0	1/1	0	1/2
	Computer gen written material (5)			5		3/4	3/4	0	0/1	0	4/5
	**Total**			**7**		**3/5**	**5/6**	**0**	**1/2**	**0**	**5/7**
**Lay worker (6)**											
	Group education (5)	3	1		1	0	2/3	0	2/4	1/1	3/5
	Mixed intervention (1)	1				1/1	1/1	0	1/1	0	0/1
	**Total**	**4**	**1**		**1**	**1/1**	**3/4**	**0**	**3/4**	**1/1**	**3/6**
**Other (7)**											
	Mixed intervention (1)	1				0/1	1/1	0/1	0/1	0	1/1
	Telephone (1)		1			0	0	0	0	0	1/1
	Written (5)		3	2		0	3/3	0	3/5	0/1	4/5
	**Total**	**1**	**4**	**2**		**0/1**	**4/4**	**0/1**	**3/6**	**0/1**	**6/7**
**MDT (11)**											
	Group education (5)	2	3			0/1	3/3	0	2/2	1/2	5/5
	Mixed intervention (5)	2	2	1		1/1	1/2	0	1/2	2/2	4/5
	Motivational interviewing and counselling (1)		1			0	0	0	1/1	0	1/1
	**Total**	**4**	**6**	**1**		**1/2**	**4/5**	**0**	**4/5**	**3/4**	**10/11**

**Table 5:** Summary of included studies

**Reference**	**Intervention**	**Study type and quality**	**SNAPW outcomes**	**Health literacy outcomes**
**Authors:** Adolfsson ET, Walker-EngstrÃm ML, Smide B, Wikblad K [36]	Patient education in type 2 diabetes-A randomized controlled 1-year follow-up study	**Study type:** Randomised Controlled Trial	**Smoking:** N/A	**Patient knowledge**: Yes
	**Intervention type:** Group education	**Quality rating:** High	**Nutrition:** N/A	**Patient skills:** N/A
	**Description:** Group empowerment sessions	**Quality score:** 2.66	**Alcohol:** N/A	**Self efficacy**: No
	**Intensity:** 4–5 sessions over 7 months		**Physical activity:** N/A	**Stage of change:** N/A
	**Intensity score:** M		**Weight:** No	
	**Provider:** Multi-disciplinary team (GP, educator)			**Patient activation**: N/A
				**Shared decision making:** N/A
				**Other health literacy outcome:** N/A
**Authors:** Aldana SG, Greenlaw RL, Diehl HA, Salberg A, Merrill RM, Ohmine S, et al [37]	Effects of an intensive diet and physical activity modification program on the health risks of adults	**Study type:** Randomised Controlled Trial	**Smoking:** N/A	**Patient knowledge**: Yes
	**Intervention type:** Group education	**Quality rating:** High	**Nutrition:** Yes	**Patient skills:** N/A
	**Description:** Group education with participants following preset dietary goals	**Quality score:** 3.0	**Alcohol:** N/A	**Self efficacy**: N/A
	**Intensity:** 40 h over a 4-week period		**Physical activity:** Yes	**Stage of change:** N/A
	**Intensity score:** M		**Weight:** Yes	**Patient activation**: N/A
	**Provider:** Multi-disciplinary team (GP, educator)			**Shared decision making:** N/A
				**Other health literacy outcome:** N/A
**Authors:** Brassington GS, Atienza AA, Perczek RE, DiLorenzo TM, King AC [38]	Intervention-Related Cognitive Versus Social	**Study type:** Randomised Trial	**Smoking:** N/A	**Patient knowledge**: N/A
	Mediators of Exercise Adherence in the Elderly	**Quality rating:** Low	**Nutrition:** N/A	**Patient skills:** N/A
	**Intervention type:** Telephone		**Alcohol:** N/A	**Self efficacy**: Yes
	**Description:** Telephone Counselling + exercise logs		**Physical activity:** Yes	**Stage of change:** N/A
	**Intensity:** exercise prescription, telephone exercise counseling to promote exercise adherence for 12 months, and attended 6- and 12-month assessment visits		**Weight:** N/A	**Patient activation**: N/A
	**Intensity score:** H	**Quality score:** 1.83		**Shared decision making:** N/A
	**Provider:** Educator			**Other health literacy outcome:** N/A
**Authors:** de Vries H, Kremers SP, Smeets T, Brug J, Eijmael K [53]	The effectiveness of tailored feedback and action plans in an intervention addressing multiple health behaviors	**Study type:** Randomised Controlled Trial	**Smoking:** No	**Patient knowledge**: N/A
	**Intervention type:** Written material	**Quality rating:** Medium	**Nutrition:** Yes	**Patient skills:** N/A
	**Description:** 3 iterative tailored feedback letters	**Quality score:** 2.0	**Alcohol:** N/A	**Self efficacy**: Yes
	**Intensity:** 3 iterative feedback letters		**Physical activity:** Yes	**Stage of change:** N/A
	**Intensity score:** L		**Weight:** N/A	**Patient activation**: N/A
	**Provider:** Other			**Shared decision making:** N/A
				**Other health literacy outcome:** N/A
**Authors:** Dunn AL, Marcus BH, Kampert JB, Garcia ME, Kohl HW, Blair SN [39]	Reduction in cardiovascular disease risk factors: 6-month results from Project Active	**Study type:** Randomised Clinical Trial	**Smoking:** N/A	**Patient knowledge**: Yes
	**Intervention type:** Motivational interviewing and counseling	**Quality rating:** Medium	**Nutrition:** N/A	**Patient skills:** N/A
	**Description:** Cognitive & behavioural strategies + Motivational Interviewing - advised to accumulate at least 30 min of moderate intensity physical activity on most days of the week, tailored to their lifestyle	**Quality score:** 2.33	**Alcohol:** N/A	**Self efficacy**: Yes
	**Intensity:** small groups that met for an hour 1 night a week for the first 16 weeks, and then every other week for weeks 17 to 24,		**Physical activity:** Yes	**Stage of change:** No
	**Intensity score:** H		**Weight:** N/A	**Patient activation**: N/A
	**Provider:** Educator			**Shared decision making:** N/A
				**Other health literacy outcome:** N/A
**Authors:** Efraimsson EÃ, Hillervik C, Ehrenberg A [40]	Effects of COPD self-care management education at a nurse-led primary health care clinic	**Study type:** Randomised Controlled Trial	**Smoking:** Yes	**Patient knowledge**: Yes
	**Intervention type:** Motivational interviewing and counseling	**Quality rating:** Low	**Nutrition:** N/A	**Patient skills:** N/A
	**Description:** Disease education (COPD) & counseling on RF	**Quality score:** 1.83	**Alcohol:** N/A	**Self efficacy**: N/A
	**Intensity:** 12–20 weeks		**Physical activity:** Yes	**Stage of change:** N/A
	**Intensity score:** H		**Weight:** N/A	**Patient activation**: N/A
	**Provider:** Nurse			**Shared decision making:** N/A
				**Other health literacy outcome:** N/A
**Authors:** Goldstein MG, Pinto BM, Marcus BH, Lynn H, Jette AM, Rakowski W, et al [32]	Physician-based physical activity counseling for middle-aged and older adults: a randomized trial	**Study type:** Randomised Controlled Trial	**Smoking:** N/A	**Patient knowledge**: N/A
	**Intervention type:** Motivational interviewing and counseling	**Quality rating:** Medium	**Nutrition:** N/A	**Patient skills:** N/A
	**Description:** Individualised motivational counseling, goal setting + PA prescription + mailed monthly materials	**Quality score:** 2.33	**Alcohol:** N/A	**Self efficacy**: N/A
	**Intensity:** 5 mins brief counseling with mailed monthly materials		**Physical activity:** No	**Stage of change:** No
	**Intensity score:** L		**Weight:** N/A	**Patient activation**: N/A
	**Provider:** Doctor			**Shared decision making:** N/A
				**Other health literacy outcome:** N/A
**Authors:** Hartman TJ, McCarthy PR, Park RJ, Schuster E, Kushi LH [54]	Results of a community-based low-literacy nutrition education program	**Study type:** Randomised Trial	**Smoking:** N/A	**Patient knowledge**: N/A
	**Intervention type:** Group education	**Quality rating:** Medium	**Nutrition:** Yes	**Patient skills:** N/A
	**Description:** 10 sessions and written information	**Quality score:** 2.5	**Alcohol:** N/A	**Self efficacy**: No
	**Intensity:** 10 sessions		**Physical activity:** N/A	**Stage of change:** N/A
	**Intensity score:** H		**Weight:** N/A	**Patient activation**: N/A
	**Provider:** Lay worker			**Shared decision making:** N/A
				**Other health literacy outcome:** N/A
**Authors:** Kloek GC, van Lenthe FJ, van Nierop PWM, Koelen MA, Mackenbach JP [55]	Impact evaluation of a Dutch community intervention to improve health-related behaviour in deprived neighbourhoods	**Study type:** Quasi experimental	**Smoking:** No	**Patient knowledge**: Yes
	**Intervention type:** Mixed intervention	**Quality rating:** Medium	**Nutrition:** Yes	**Patient skills:** N/A
	**Description:** Group education + community development (one off special events)	**Quality score:** 2.16	**Alcohol:** No	**Self efficacy**: Yes
	**Intensity:** 2 year intervention		**Physical activity:** No	**Stage of change:** Yes
	**Intensity score:** H		**Weight:** N/A	**Patient activation**: N/A
	**Provider:** Other			**Shared decision making:** N/A
				**Other health literacy outcome:** No
**Authors:** Lorig KR, Ritter PL, Jacquez A [56]	Outcomes of border health Spanish/English Chronic Disease Self-management Programs	**Study type:** Before and After Study	**Smoking:** N/A	**Patient knowledge**: N/A
	**Intervention type:** Group education	**Quality rating:** Medium	**Nutrition:** Yes	**Patient skills:** N/A
	**Description:** CDSMP	**Quality score:** 2.16	**Alcohol:** N/A	**Self efficacy**: Yes
	**Intensity:** 2.5 h sessions over 6 weeks - total 14 h		**Physical activity:** Yes	**Stage of change:** N/A
	**Intensity score:** H		**Weight:** N/A	**Patient activation**: N/A
	**Provider:** Lay worker			**Shared decision making:** N/A
				**Other health literacy outcome:** N/A
**Authors:** Nies MA, Artinian NT, Schim SM, Vander Wal JS, Sherrick-Escamilla S [57]	Effects of lay health educator interventions on activity, diet, and health risks in an urban Mexican American community	**Study type:** Before and After Study	**Smoking:** N/A	**Patient knowledge**: No
	**Intervention type:** Group education	**Quality rating:** Low	**Nutrition:** No	**Patient skills:** N/A
	**Description:** Health promotion train the trainer sessions in the community	**Quality score:** 1.66	**Alcohol:** N/A	**Self efficacy**: N/A
	**Intensity:** 2–6 h train the trainer sessions and 3x1h sessions for community		**Physical activity:** No	**Stage of change:** N/A
	**Intensity score:** M		**Weight:** N/A	**Patient activation**: N/A
	**Provider:** Lay worker			**Shared decision making:** N/A
				**Other health literacy outcome:** N/A
**Authors:** Norris SL, Grothaus LC, Buchner DM, Pratt M [33]	Effectiveness of physician-based assessment and counseling for exercise in a staff model HMO	**Study type:** Randomised Controlled Trial	**Smoking:** N/A	**Patient knowledge**: N/A
	**Intervention type:** Motivational interviewing and counseling	**Quality rating:** High	**Nutrition:** N/A	**Patient skills:** N/A
	**Description:** One counselling session, written PA prescription and one follow up call. A subset received booster telephone calls at 2, 3 and 4 months and postcard reminders at 2, 3, 4, and 5 months. (no significant difference between groups in PA levels)	**Quality score:** 2.66	**Alcohol:** N/A	**Self efficacy**: No
	**Intensity:** One counselling session, written PA prescription and one follow up call		**Physical activity:** Yes	**Stage of change:** N/A
	**Intensity score:** L		**Weight:** N/A	**Patient activation**: N/A
	**Provider:** Doctor			**Shared decision making:** N/A
				**Other health literacy outcome:** No
**Authors:** Ridgeway NA, Harvill DR, Harvill LM, Falin TM, Forester GM, Gose OD [41]	Improved control of type 2 diabetes mellitus: A practical education/behavior modification program in a primary care clinic	**Study type:** Randomised Controlled Trial	**Smoking:** N/A	**Patient knowledge**: Yes
	**Intervention type:** Mixed intervention	**Quality rating:** Medium	**Nutrition:** N/A	**Patient skills:** N/A
	**Description:** Group education mixed with individual education sessions	**Quality score:** 2.16	**Alcohol:** N/A	**Self efficacy**: N/A
	**Intensity:** 12 weekly group sessions, 1 individual session and 6 bi-weekly sessions		**Physical activity:** N/A	**Stage of change:** N/A
	**Intensity score:** H		**Weight:** Yes	**Patient activation**: N/A
	**Provider:** Multi-disciplinary team (Nurse, dietician, GP)			**Shared decision making:** N/A
				**Other health literacy outcome:** N/A
**Authors:** Sadur CN, Moline N, Costa M, Michalik D, Mendlowitz D, Roller S, et al [42]	Diabetes management in a health maintenance organization: Efficacy of care management using cluster visits	**Study type:** Clustered Randomised Controlled Trial	**Smoking:** N/A	**Patient knowledge**: N/A
	**Intervention type:** Group education	**Quality rating:** Medium	**Nutrition:** Yes	**Patient skills:** N/A
	**Description:** Group education + 1 additional individual session	**Quality score:** 2.33	**Alcohol:** N/A	**Self efficacy**: Yes
	**Intensity:** 10–18 patients/month for 6 months		**Physical activity:** N/A	**Stage of change:** N/A
	**Intensity score:** H		**Weight:** N/A	**Patient activation**: N/A
	**Provider:** Multi-disciplinary team (Psychologist, educator, dietician, pharmacist)			**Shared decision making:** N/A
				**Other health literacy outcome:** N/A
**Authors:** Van Sluijs EMF, Van Poppel MNM, Twisk JWR, Van Mechelen W [43]	Physical activity measurements affected participants’ behavior in a randomized controlled trial	**Study type:** Randomised Controlled Trial	**Smoking:** N/A	**Patient knowledge**: No
	**Intervention type:** Motivational interviewing and counseling	**Quality rating:** Medium	**Nutrition:** N/A	**Patient skills:** N/A
	**Description:** Individual physician tailored counseling + 2 phone calls (5–10mins) + follow up consultation	**Quality score:** 2.0	**Alcohol:** N/A	**Self efficacy**: No
	**Intensity:** 10 min initial consult, 5–10 min phone call at 2 weeks, 10 min consult at 4 weeks and phone call 8 weeks after 2nd consult		**Physical activity:** Yes	**Stage of change:** Yes
	**Intensity score:** M		**Weight:** N/A	**Patient activation**: N/A
	**Provider:** Multi-disciplinary team (GP, nurse, counselor)			**Shared decision making:** N/A
				**Other health literacy outcome:** No
**Authors:** Yajima S, Takano T, Nakamura K, Watanabe M [58]	Effectiveness of a community leaders’ programme to promote healthy lifestyles in Tokyo, Japan	**Study type:** Quasi experimental	**Smoking:** No	**Patient knowledge**: N/R
	**Intervention type:** Mixed intervention	**Quality rating:** Medium	**Nutrition:** Yes	**Patient skills:** N/A
	**Description:** Range of activities by lay community members to be health promotion leaders	**Quality score:** 2.0	**Alcohol:** N/A	**Self efficacy**: N/A
	**Intensity:** 2 year program		**Physical activity:** Yes	**Stage of change:** N/A
	**Intensity score:** H		**Weight:** N/A	**Patient activation**: N/R
	**Provider:** Lay worker			**Shared decision making:** N/A
				**Other health literacy outcome:** Yes
**Authors:** Boylan MJ, Renier CM, Knuths JS, Haller IV [44]	Preventing cardiovascular disease in women: an intervention-control randomized study	**Study type:** Randomised Trial	**Smoking:** No	**Patient knowledge**: N/A
	**Intervention type:** Group education	**Quality rating:** Medium	**Nutrition:** N/A	**Patient skills:** N/A
	**Description:** Lifestyle intervention program including individualized review, information, classes and follow up phone calls	**Quality score:** 2.0	**Alcohol:** N/A	**Self efficacy**: Yes
	**Intensity:** 12 months		**Physical activity:** N/A	**Stage of change:** N/A
	**Intensity score:** H		**Weight:** N/A	**Patient activation**: N/A
	**Provider:** Educator			**Shared decision making:** N/A
				**Other health literacy outcome:** N/A
**Authors:** Jimmy G, Martin BW [59]	Implementation and effectiveness of a primary care based physical activity counselling scheme	**Study type:** Randomised Trial	**Smoking:** N/A	**Patient knowledge**: N/A
	**Intervention type:** Mixed intervention	**Quality rating:** Medium	**Nutrition:** N/A	**Patient skills:** N/A
	**Description:** Individual counseling & feedback on stage of change + take home leaflets + offer of referral to PA specialist (at ¼ of cost) + follow up session computer assisted tool (PA & motivation assessment) + telephone reminders (3)	**Quality score:** 2.33	**Alcohol:** N/A	**Self efficacy**: N/A
	**Intensity:** Feedback, information 45 min counselling session		**Physical activity:** No	**Stage of change:** No
	**Intensity score:** M		**Weight:** N/A	**Patient activation**: N/A
	**Provider:** Doctor			**Shared decision making:** N/A
				**Other health literacy outcome:** N/A
**Authors:** Kreuter MW, Strecher VJ [60]	Do tailored behavior change messages enhance the effectiveness of health risk appraisal? Results from a randomized trial	**Study type:** Randomised Controlled Trial	**Smoking:** No	**Patient knowledge**: N/A
	**Intervention type:** Written material	**Quality rating:** High	**Nutrition:** No	**Patient skills:** N/A
	**Description:** individually-tailored behavior change information with health risk assessment mailed	**Quality score:** 2.66	**Alcohol:** N/A	**Self efficacy**: N/A
	**Intensity:** 1 off risk information sheet and 1 individually tailored behavior change info		**Physical activity:** No	**Stage of change:** No
	**Intensity score:** L		**Weight:** N/A	**Patient activation**: N/A
	**Provider:** Computer			**Shared decision making:** N/A
				**Other health literacy outcome:** N/A
**Authors:** Miller CK, Edwards L, Kissling G, Sanville L [45]	Evaluation of a theory-based nutrition intervention for older adults with diabetes mellitus	**Study type:** Randomised Controlled Trial	**Smoking:** N/A	**Patient knowledge**: Yes
	**Intervention type:** Group education	**Quality rating:** Medium	**Nutrition:** N/A	**Patient skills:** N/A
	**Description:** 10 group education sessions	**Quality score:** 2.33	**Alcohol:** N/A	**Self efficacy**: Yes
	**Intensity:** 10 weekly group sessions		**Physical activity:** N/A	**Stage of change:** N/A
	**Intensity score:** H		**Weight:** N/A	**Patient activation**: N/A
	**Provider:** Dietician			**Shared decision making:** N/A
				**Other health literacy outcome:** N/A
**Authors:** Oenema A, Brug J, Dijkstra A, de Weerdt I, de Vries H [61]	Efficacy and use of an internet-delivered computer-tailored lifestyle intervention, targeting saturated fat intake, physical activity and smoking cessation: a randomized controlled trial	**Study type:** Randomised Controlled Trial	**Smoking:** No	**Patient knowledge**: N/A
	**Intervention type:** Computer	**Quality rating:** Medium	**Nutrition:** Yes	**Patient skills:** N/A
	**Description:** Internet-delivered, computer-tailored lifestyle intervention	**Quality score:** 2.5	**Alcohol:** N/A	**Self efficacy**: N/A
	**Intensity:** 1 month		**Physical activity:** Yes	**Stage of change:** No
	**Intensity score:** L		**Weight:** N/A	**Patient activation**: N/A
	**Provider:**			**Shared decision making:** N/A
	Computer			**Other health literacy outcome:** N/A
**Authors:** Ogden J, Hoppe R [62]	The relative effectiveness of two styles of educational package to change practice nurses’ management of obesity	**Study type:** Before and After Study	**Smoking:** N/A	**Patient knowledge**: N/A
	**Intervention type:** Motivational interviewing and counseling	**Quality rating:** Medium	**Nutrition:** N/A	**Patient skills:** Yes
	**Description:** Individual consultation + advice by nurse	**Quality score:** 2.0	**Alcohol:** N/A	**Self efficacy**: N/A
	**Intensity:** One consultation		**Physical activity:** N/A	**Stage of change:** N/A
	**Intensity score:** L		**Weight:** No	**Patient activation**: N/A
	**Provider:** Nurse			**Shared decision making:** N/A
				**Other health literacy outcome:** N/A
**Authors:** Simmons D, Voyle JA, Fout F, Feot S, Leakehe L [63]	Tale of two churches: Differential impact of a church-based diabetes control programme among Pacific Islands people in New Zealand	**Study type:** Non-Randomised Controlled Trial	**Smoking:** N/A	**Patient knowledge**: Yes
	**Intervention type:** Group education	**Quality rating:** High	**Nutrition:** N/A	**Patient skills:** N/A
	**Description:** Tailored activities by language & culture relevance & cooking classes	**Quality score:** 2.66	**Alcohol:** N/A	**Self efficacy**: N/A
	**Intensity:** NR		**Physical activity:** No	**Stage of change:** Yes
	**Intensity score:** NR		**Weight:** Yes	**Patient activation**: N/A
	**Provider:** Lay worker			**Shared decision making:** N/A
				**Other health literacy outcome:** No
**Authors:** Ryan A, Smith C [64]	Change for Life/Cambia tu vida: a health promotion program based on the stages of change model for African descendent and Latino adults in New Hampshire	**Study type:** Before and After Study	**Smoking:** N/A	**Patient knowledge**: N/A
	**Intervention type:** Group education	**Quality rating:** Medium	**Nutrition:** N/A	**Patient skills:** N/A
	**Description:** Group education targeting participants stage of change + culturally appropriate written resources + decision tree	**Quality score:** 2.33	**Alcohol:** N/A	**Self efficacy**: N/A
	**Intensity:** six 2-h classes held weekly, followed by periodic group support meetings after the series of classes is completed		**Physical activity:** N/A	**Stage of change:** Yes
	**Intensity score:** M		**Weight:** N/A	**Patient activation**: N/A
	**Provider:** Multi-disciplinary team (Researcher, lay worker)			**Shared decision making:** N/A
				**Other health literacy outcome:** N/A
**Authors:** Steptoe A, Rink E, Kerry S [65]	Psychosocial predictors of changes in physical activity in overweight sedentary adults following counseling in primary care	**Study type:** Randomised Controlled Trial	**Smoking:** No	**Patient knowledge**: N/A
	**Intervention type:** Motivational interviewing and counseling	**Quality rating:** Medium	**Nutrition:** N/A	**Patient skills:** N/A
	**Description:** Individual brief counseling by a nurse (1–3 sessions)	**Quality score:** 2.33	**Alcohol:** N/A	**Self efficacy**: Yes
	**Intensity:** 1–3 sessions		**Physical activity:** Yes	**Stage of change:** Yes
	**Intensity score:** L		**Weight:** N/A	**Patient activation**: N/A
	**Provider:** Nurse			**Shared decision making:** N/A
				**Other health literacy outcome:** No
**Authors:** Toobert DJ, Glasgow RE, Strycker LA, Barrera M, Ritzwoller DP, Weidner G [46]	Long-term effects of the Mediterranean lifestyle program: a randomized clinical trial for postmenopausal women with type 2 diabetes (Provisional abstract)	**Study type:** Randomised Controlled Trial	**Smoking:** No	**Patient knowledge**: N/A
	**Intervention type:** Group education	**Quality rating:** Medium	**Nutrition:** Yes	**Patient skills:** N/A
	**Description:** 2.5 day non residential education + weekly meetings for 6 months with small group support	**Quality score:** 2.33	**Alcohol:** N/A	**Self efficacy**: Yes
	**Intensity:** 2.5 day non residential education + weekly meetings for 6 months with small group support		**Physical activity:** Yes	**Stage of change:** N/A
	**Intensity score:** H		**Weight:** N/A	**Patient activation**: N/A
	**Provider:** Multi-disciplinary team (Dietician, exercise physiologist, educator, lay worker)			**Shared decision making:** N/A
				**Other health literacy outcome:** Yes
**Authors:** Hoffman AM, Redding CA, Goldberg D, Añel D, Prochaska JO, Meyer PM, et al [66]	Computer expert systems for African-American smokers in physicians offices: A feasibility study	**Study type:** Randomised Trial	**Smoking:** Yes	**Patient knowledge**: N/A
	**Intervention type:** Mixed intervention	**Quality rating:** Medium	**Nutrition:** N/A	**Patient skills:** N/A
	**Description:** Computer feedback with nurse + stage based RF manual + 3–6 min audio-tapes + stress management exercise instructions	**Quality score:** 2.0	**Alcohol:** N/A	**Self efficacy**: N/A
	**Intensity:** Interactive computer sessions (number uncertain) and brief audiotapes		**Physical activity:** N/A	**Stage of change:** Yes
	**Intensity score:** M		**Weight:** N/A	**Patient activation**: N/A
	**Provider:** Computer			**Shared decision making:** N/A
				**Other health literacy outcome:** N/A
**Authors:** Wolf RL, Lepore SJ, Vandergrift JL, Basch CE, Yaroch AL [67]	Tailored telephone education to promote awareness and adoption of fruit and vegetable recommendations among urban and mostly	**Study type:** Randomised Controlled Trial	**Smoking:** N/A	**Patient knowledge**: Yes
	**Intervention type:** Telephone	**Quality rating:** High	**Nutrition:** N/A	**Patient skills:** N/A
	**Description:** Telephone individual education & mailed brochure	**Quality score:** 2.66	**Alcohol:** N/A	**Self efficacy**: N/A
	**Intensity:** 2 calls and mailed brochure		**Physical activity:** N/A	**Stage of change:** Yes
	**Intensity score:** M		**Weight:** N/A	**Patient activation**: N/A
	**Provider:** Other			**Shared decision making:** N/A
				**Other health literacy outcome:** N/A
**Authors:** Swerissen H, Belfrage J, Weeks A, Jordan L, Walker C, Furler J, et al [68]	A Randomized Controlled Trial of Financial Incentives for Smoking Cessation	**Study type:** Randomised Controlled Trial	**Smoking:** N/A	**Patient knowledge**: N/A
	**Intervention type:** Group education	**Quality rating:** Medium	**Nutrition:** N/A	**Patient skills:** N/A
	**Description:** Language specific SM Program + audiocassette + program booklet	**Quality score:** 2.0	**Alcohol:** N/A	**Self efficacy**: Yes
	**Intensity:** 6 weekly 2.5 h sessions. 20 h training for peer leaders		**Physical activity:** Yes	**Stage of change:** N/A
	**Intensity score:** H		**Weight:** N/A	**Patient activation**: N/A
	**Provider:** Lay worker			**Shared decision making:** N/A
				**Other health literacy outcome:** N/A
**Authors:** Campbell J, Aday RH [69]	Improving dietary behavior: the effectiveness of tailored messages in primary care settings	**Study type:** Randomised Controlled Trial	**Smoking:** N/A	**Patient knowledge**: N/A
	**Intervention type:** Written material	**Quality rating:** High	**Nutrition:** Yes	**Patient skills:** N/A
	**Description:** Computer generated tailored nutrition newsletters & profile feedback related to stage of change	**Quality score:** 3.0	**Alcohol:** N/A	**Self efficacy**: N/A
	**Intensity:** one-time, mailed nutrition information packet		**Physical activity:** N/A	**Stage of change:** Yes
	**Intensity score:** L		**Weight:** N/A	**Patient activation**: N/A
	**Provider:** Computer			**Shared decision making:** N/A
				**Other health literacy outcome:** Yes
**Authors:** Collins R, Lee RE, Albright CL, King AC [47]	Ready to be Physically Active? The Effects of a Course Preparing Low-Income Multiethnic Women to be more Physically Active	**Study type:** Before and After Study	**Smoking:** N/A	**Patient knowledge**: Yes
	**Intervention type:** Group education	**Quality rating:** Medium	**Nutrition:** N/A	**Patient skills:** N/A
	**Description:** Group Education + culturally sensitive curriculum	**Quality score:** 2.16	**Alcohol:** N/A	**Self efficacy**: No
	**Intensity:** 10 mth intervention		**Physical activity:** Yes	**Stage of change:** Yes
	**Intensity score:** H		**Weight:** N/A	**Patient activation**: N/A
	**Provider:** Educator			**Shared decision making:** N/A
				**Other health literacy outcome:** Yes
**Authors:** Gladys Block PW, Rochelle Mandel, Diane Metz, Mary L Fujii, Nancy Feldman, and Barbara Sutherland [70]	A Randomized Trial of the Little by Little CD-ROM: Demonstrated Effectiveness in Increasing Fruit and Vegetable Intake in a Low-income Population	**Study type:** Randomised Trial	**Smoking:** N/A	**Patient knowledge**: N/A
	**Intervention type:** Computer	**Quality rating:** Medium	**Nutrition:** Yes	**Patient skills:** N/A
	**Description:** Self Guided interactive computer program + goal setting + handouts	**Quality score:** 2.33	**Alcohol:** N/A	**Self efficacy**: N/A
	**Intensity:** CD ROM and 2 reminder phone calls		**Physical activity:** N/A	**Stage of change:** Yes
	**Intensity score:** L		**Weight:** N/A	**Patient activation**: N/A
	**Provider:** Computer			**Shared decision making:** N/A
				**Other health literacy outcome:** N/A
**Authors:** Koffman DM BT, Mosca L, Redberg R, Schmid T, Wattigney WA [71]	An evaluation of Choose to Move 1999: an American Heart Association physical activity program for women.	**Study type:** Before and After Study	**Smoking:** N/A	**Patient knowledge**: Yes
	**Intervention type:** Written material	**Quality rating:** Low	**Nutrition:** Yes	**Patient skills:** N/A
	**Description:** written materials + Postcards + Email + Newsletter	**Quality score:** 1.5	**Alcohol:** N/A	**Self efficacy**: N/A
	**Intensity:** 12-week, mail-mediated lifestyle intervention program		**Physical activity:** Yes	**Stage of change:** N/A
	**Intensity score:** M		**Weight:** N/A	**Patient activation**: N/A
	**Provider:** Other			**Shared decision making:** N/A
				**Other health literacy outcome:** N/A
**Authors:** Marcus BH BB, Pinto BM, Forsyth LH, Roberts MB, Traficante RM [72]	Efficacy of an individualized, motivationally-tailored physical activity intervention	**Study type:** Randomised Trial	**Smoking:** N/A	**Patient knowledge**: N/A
	**Intervention type:** Written material	**Quality rating:** Medium	**Nutrition:** N/A	**Patient skills:** N/A
	**Description:** Tailored counseling messages using “computer expert system” + motivationally matched manuals + feedback on progress were mailed to subjects	**Quality score:** 2.16	**Alcohol:** N/A	**Self efficacy**: Yes
	**Intensity:** NR		**Physical activity:** Yes	**Stage of change:** Yes
	**Intensity score:** M		**Weight:** N/A	**Patient activation**: N/A
	**Provider:** Other			**Shared decision making:** N/A
				**Other health literacy outcome:** N/A
**Authors:** Prochaska JO, Velicer WF, Redding C, Rossi JS, Goldstein M, DePue J, et al [73]	Stage-based expert systems to guide a population of primary care patients to quit smoking, eat healthier, prevent skin cancer, and receive regular mammograms	**Study type:** Randomised Controlled Trial	**Smoking:** Yes	**Patient knowledge**: N/A
	**Intervention type:** Written material	**Quality rating:** Medium	**Nutrition:** Yes	**Patient skills:** N/A
	**Description:** Mailed computer generated profile reports (stage of change, use of change processes, pros & cons of changing) + self help manual + strategies on how to progress stages	**Quality score:** 2.33	**Alcohol:** N/A	**Self efficacy**: N/A
	**Intensity:** 3 reports mailed for each risk factor		**Physical activity:** N/A	**Stage of change:** Yes
	**Intensity score:** L		**Weight:** N/A	**Patient activation**: N/A
	**Provider:** Computer			**Shared decision making:** N/A
				**Other health literacy outcome:** N/A
**Authors:** Prochaska JO, Velicer WF, Rossi JS, Redding CA, Greene GW, Rossi SR, et al [74]	Multiple Risk Expert Systems Interventions: Impact of Simultaneous Stage-Matched Expert System Interventions for Smoking, High-Fat Diet, and Sun Exposure in a Population of Parents	**Study type:** Randomised Controlled Trial	**Smoking:** Yes	**Patient knowledge**: N/A
	**Intervention type:** Written material	**Quality rating:** Medium	**Nutrition:** Yes	**Patient skills:** N/A
	**Description:** Mailed computer generated profile reports (stage of change, use of change processes, pros & cons of changing) + self help manual + strategies on how to progress stages	**Quality score:** 2.16	**Alcohol:** N/A	**Self efficacy**: N/A
	reports for each of their relevant behaviors at 0, 6, and 12 months as well as a multiple behavior manual.		**Physical activity:** N/A	**Stage of change:** Yes
	**Intensity:** Received 3 reports per year		**Weight:** N/A	**Patient activation**: N/A
	**Intensity score:** L			**Shared decision making:** N/A
	**Provider:** Computer			**Other health literacy outcome:** N/A
**Authors:** Beresford SA SJC, A R Kristal, D Lazovich, Z Feng and E H Wagner [34]	A dietary intervention in primary care practice: the Eating Patterns Study.	**Study type:** Randomised Controlled Trial	**Smoking:** N/A	**Patient knowledge**: N/A
	**Intervention type:** Written material	**Quality rating:** High	**Nutrition:** Yes	**Patient skills:** No
	**Description:** Self help materials	**Quality score:** 2.66	**Alcohol:** N/A	**Self efficacy**: N/A
	**Intensity:** Low intensity – time not stated		**Physical activity:** N/A	**Stage of change:** No
	**Intensity score:** L		**Weight:** N/A	**Patient activation**: N/A
	**Provider:** Doctor			**Shared decision making:** N/A
				**Other health literacy outcome:** N/A
**Authors:** Marcus B H Emmons KM, Simkin-Silverman L R, Linnan L A, Taylor E R, Bock B C, Roberts M B, Rossi J S, Abrams D B [75]	Evaluation of motivationally tailored vs standard self-help physical activity interventions at the workplace	**Study type:** Randomised Trial	**Smoking:** N/A	**Patient knowledge**: N/A
	**Intervention type:** Written material	**Quality rating:** Medium	**Nutrition:** N/A	**Patient skills:** N/A
	**Description:** Repeated mailing (3 times) Self-help manuals + motivational messages related to stage of change	**Quality score:** 2.0	**Alcohol:** N/A	**Self efficacy**: N/A
	**Intensity:** 3 lots of written material		**Physical activity:** No	**Stage of change:** Yes
	**Intensity score:** L		**Weight:** N/A	**Patient activation**: N/A
	**Provider:** Other			**Shared decision making:** N/A
				**Other health literacy outcome:** N/A
**Authors:** Graham-Clarke P, Oldenburg B [76]	The effectiveness of a general-practice-based physical activity intervention on patient physical activity status	**Study type:** Randomised Trial	**Smoking:** N/A	**Patient knowledge**: N/A
	**Intervention type:** Motivational interviewing and counseling	**Quality rating:** High	**Nutrition:** N/A	**Patient skills:** N/A
	**Description:** Lifestyle counseling - Fresh Start Program by Heart Foundation	**Quality score:** 2.66	**Alcohol:** N/A	**Self efficacy**: N/A
	**Intensity:** NR		**Physical activity:** No	**Stage of change:** Yes
	**Intensity score:** NR		**Weight:** N/A	**Patient activation**: N/A
	**Provider:** Doctor			**Shared decision making:** N/A
				**Other health literacy outcome:** No
**Authors:** O’Loughlin, Jennifer Paradis, Gilles Meshefedjian, Garbis Kishchuk, Natalie [77]	Evaluation of an 8-Week Mailed Healthy-Weight Intervention	**Study type:** Randomised Controlled Trial	**Smoking:** N/A	**Patient knowledge**: Unsure
	**Intervention type:** Written material	**Quality rating:** Medium	**Nutrition:** Yes	**Patient skills:** N/A
	**Description:** 8-Week Mailed Healthy-Weight Intervention	**Quality score:** 2.33	**Alcohol:** N/A	**Self efficacy**: No
	**Intensity:** 18 pamphlets mailed over 8 weeks		**Physical activity:** Unsure	**Stage of change:** No
	**Intensity score:** M		**Weight:** No	**Patient activation**: No
	**Provider:** Other			**Shared decision making:** N/A
				**Other health literacy outcome:** N/A
**Authors:** Winkleby MA, Howard-Pitney B, Albright CA, Bruce B, Kraemer HC, Fortmann SP [48]	Predicting achievement of a low-fat diet: a nutrition intervention for adults with low literacy skills	**Study type:** Randomised Controlled Trial	**Smoking:** N/A	**Patient knowledge**: Yes
	**Intervention type:** Mixed intervention	**Quality rating:** High	**Nutrition:** Yes	**Patient skills:** N/A
	**Description:** Group education (Stanford Nutrition Action Program) + multiple mail/telephone follow up calls	**Quality score:** 2.66	**Alcohol:** N/A	**Self efficacy**: Yes
	**Intensity:** six or seven 60-min sessions classes		**Physical activity:** N/A	**Stage of change:** N/A
	**Intensity score:** H		**Weight:** No	**Patient activation**: N/A
	**Provider:** Educator			**Shared decision making:** N/A
				**Other health literacy outcome:** N/A
**Authors:** Agurs-Collins, TD, Kumanyika, SK, Ten Have, TR, Adams-Campbell, LL [49]	A randomised controlled trial of weight reduction and exercise for diabetes management in older African-American subjects.	**Study type:** Randomised Controlled Trial	**Smoking:** N/A	**Patient knowledge**: Unsure
	**Intervention type:** Mixed intervention	**Quality rating:** Medium	**Nutrition:** No	**Patient skills:** N/A
	**Description:** 6 group sessions, 1 individualised counseling + diary	**Quality score:** 2.16	**Alcohol:** N/A	**Self efficacy**: N/A
	**Intensity:** 12 weekly group sessions, 1 individual session and 6 bi-weekly group sessions		**Physical activity:** No	**Stage of change:** Unsure Not reported
	**Intensity score:** H		**Weight:** Yes	**Patient activation**: N/A
	**Provider:** Multi-disciplinary team (Dietician, exercise physiologist)			**Shared decision making:** N/A
				**Other health literacy outcome:** N/A
**Authors:** Butler, CC, Rollnick, S, Cohen, D et al, [78]	Motivational consulting versus brief advice for smokers in general practice: a randomized trial	**Study type:** Randomised Trial	**Smoking:** Yes	**Patient knowledge**: N/A
	**Intervention type:** Motivational interviewing and counseling	**Quality rating:** Medium	**Nutrition:** N/A	**Patient skills:** N/A
	**Description:** Motivational Counselling + patient setting targets	**Quality score:** 2.0	**Alcohol:** N/A	**Self efficacy**: N/A
	**Intensity:** NR		**Physical activity:** N/A	**Stage of change:** Yes
	**Intensity score:** NR		**Weight:** N/A	**Patient activation**: N/A
	**Provider:** Doctor			**Shared decision making:** N/A
				**Other health literacy outcome:** N/A
**Authors:** Calfas, KJ, Sallis, JF, Oldenburg, B et al, [35]	Mediators of change in physical activity following an intervention in primary care: PACE	**Study type:** Randomised Controlled Trial	**Smoking:** N/A	**Patient knowledge**: N/A
	**Intervention type:** Motivational interviewing and counseling	**Quality rating:** Medium	**Nutrition:** N/A	**Patient skills:** N/A
	**Description:** Motivational counseling + patient setting goals + activity log + examples of activities	**Quality score:** 2.33	**Alcohol:** N/A	**Self efficacy**: No
	**Intensity:** Brief intervention		**Physical activity:** Yes	**Stage of change:** Yes
	\**Intensity score:** L		**Weight:** N/A	**Patient activation**: N/A
	**Provider:**			**Shared decision making:** N/A
	Doctor			**Other health literacy outcome:** No
**Authors:** Delichatsios, HK, Hunt, MK, Lobb, R et al, [50]	EatSmart: efficacy of a multifaceted preventive nutrition intervention in clinical practice	**Study type:** Clustered	**Smoking:** N/A	**Patient knowledge**: N/A
	**Intervention type:** Mixed intervention	Randomised Controlled Trial	**Nutrition:** Yes	**Patient skills:** N/A
	**Description:** Tailored recommendations & stage matched booklets by mail + Motivational Counseling + personalized letter + physician endorsement + option of referral to counselor	**Quality rating:** Medium	**Alcohol:** N/A	**Self efficacy**: N/A
	**Intensity:** 1 mailed information, verbal endorsement by provider and 2 motivational sessions with phone counsellor. Dietitian consult offered	**Quality score:** 2.33	**Physical activity:** N/A	**Stage of change:** Yes
	**Intensity score:** M		**Weight:** N/A	**Patient activation**: N/A
	**Provider:** Multi-disciplinary team (GP, counselor)			**Shared decision making:** N/A
				**Other health literacy outcome:** N/A
**Authors:** Fries,E, Edinboro, P, McClish, D, Manion, L, Bowen, D, Beresford, SAA, Ripley, J [79]	Randomized trial of a low-intensity dietary intervention in rural residents: the Rural Physician Cancer Prevention Project.	**Study type:** Randomised Trial	**Smoking:** N/A	**Patient knowledge**: No
	**Intervention type:** Mixed intervention	**Quality rating:** Medium	**Nutrition:** Yes	**Patient skills:** N/A
	**Description:** A series of tailored feedback, brief telephone counseling + booklets	**Quality score:** 2.16	**Alcohol:** N/A	**Self efficacy**: No
	**Intensity:** Feedback, 1 phone call and 4 booklets mailed weekly		**Physical activity:** N/A	**Stage of change:** N/A
	**Intensity score:** M		**Weight:** N/A	**Patient activation**: N/A
	**Provider:** Doctor			**Shared decision making:** N/A
				**Other health literacy outcome:** No
**Authors:** Lancaster, T, Dobbie, W, Vos, K et al [51]	Randomized trial of nurse-assisted strategies for smoking cessation in primary care	**Study type:** Randomised Trial	**Smoking:** No	**Patient knowledge**: N/A
	**Intervention type:** Motivational interviewing and counseling	**Quality rating:** Medium	**Nutrition:** N/A	**Patient skills:** N/A
	**Description:** Brief advice by a doctor followed by extended counseling from a nurse	**Quality score:** 2.16	**Alcohol:** N/A	**Self efficacy**: N/A
	**Intensity:** 6 weeks		**Physical activity:** N/A	**Stage of change:** No
	**Intensity score:** M		**Weight:** N/A	**Patient activation**: N/A
	**Provider:** Nurse			**Shared decision making:** N/A
				**Other health literacy outcome:** N/A
**Authors:** Little, P, Dorward, M, Gralton, S, Hammerton, L, Pillinger, J, White, P et al. [80]	A randomised controlled trial of three pragmatic approaches to initiate increased physical activity in sedentary patients with risk factors for cardiovascular disease.	**Study type:** Randomised Trial	**Smoking:** N/A	**Patient knowledge**: N/A
	**Intervention type:** Mixed intervention	**Quality rating:** High	**Nutrition:** N/A	**Patient skills:** N/A
	**Description:** Exercise Prescription provide by GP + counseling by practice nurses and booklet	**Quality score:** 2.83	**Alcohol:** N/A	**Self efficacy**: N/A
	**Intensity:** Brief intervention with GP, 1 counseling session with nurse and material		**Physical activity:** Yes	**Stage of change:** Yes
	**Intensity score:** L		**Weight:** N/A	**Patient activation**: N/A
	**Provider:** Multi-disciplinary team (GP, nurse)			**Shared decision making:** N/A
				**Other health literacy outcome:** N/A
**Authors:** Naylor, PJ, Simmonds, G, Riddoch, C et al. [81]	Comparison of stage-matched and unmatched interventions to promote exercise behaviour in the primary care setting	**Study type:** Randomised Trial	**Smoking:** N/A	**Patient knowledge**: N/A
	**Intervention type:** Mixed intervention	**Quality rating:** Low	**Nutrition:** N/A	**Patient skills:** N/A
	**Description:** General advice and written materials or counseling and 4 staged booklets or 4 staged booklets and action planner for all groups	**Quality score:** 1.66	**Alcohol:** N/A	**Self efficacy**: No
	**Intensity:** Single contact interventions		**Physical activity:** No	**Stage of change:** Yes
	**Intensity score:** L		**Weight:** N/A	**Patient activation**: N/A
	**Provider:** Nurse			**Shared decision making:** N/A
				**Other health literacy outcome:** N/A
**Authors:** Siero, FW, Broer, J, Bemelmans, WJ, Meyboom-de Jong, BM [52]	Impact of group nutrition education and surplus value of Prochaska-based stage-matched information on health-related cognitions and on Mediterranean nutrition behavior.	**Study type:** Randomised Controlled Trial	**Smoking:** N/A	**Patient knowledge**: N/A
	**Intervention type:** Group education	**Quality rating:** Medium	**Nutrition:** Yes	**Patient skills:** N/A
	**Description:** Group education + booklets (core information)	**Quality score:** 2.16	**Alcohol:** N/A	**Self efficacy**: No
	**Intensity:** 3 sessions of 2 h each		**Physical activity:** N/A	**Stage of change:** Yes
	**Intensity score:** M		**Weight:** N/A	**Patient activation**: N/A
	**Provider:** Educator			**Shared decision making:** N/A
				**Other health literacy outcome:** Yes
**Authors:** Slama K, Redman S, Perkins J et al [82]	The effectiveness of two smoking cessation programmes for use in general practice: a randomised controlled trial.	**Study type:** Randomised Controlled Trial	**Smoking:** Yes	**Patient knowledge**: N/A
	**Intervention type:** Written material	**Quality rating:** Medium	**Nutrition:** N/A	**Patient skills:** N/A
	**Description:** Asked if a smoker and given a brochure	**Quality score:** 2.0	**Alcohol:** N/A	**Self efficacy**: N/A
	**Intensity:** Brief		**Physical activity:** N/A	**Stage of change:** N/A
	**Intensity score:** L		**Weight:** N/A	**Patient activation**: N/A
	**Provider:** Doctor			**Shared decision making:** N/A
				**Other health literacy outcome:** N/A
**Authors:** Lennox AS, Osman LM, Reiter E, Robertson R, Friend J, McCann I, Skatun D, Donnan PT [83]	Cost effectiveness of computer tailored and non-tailored smoking cessation letters in general practice: randomised controlled.	**Study type:** Randomised Controlled Trial	**Smoking:** Yes	**Patient knowledge**: N/A
	**Intervention type:** Written material	**Quality rating:** Medium	**Nutrition:** N/A	**Patient skills:** N/A
	**Description:** Tailored letter	**Quality score:** 2.16	**Alcohol:** N/A	**Self efficacy**: N/A
	**Intensity:** Brief		**Physical activity:** N/A	**Stage of change:** Yes
	**Intensity score:** L		**Weight:** N/A	**Patient activation**: N/A
	**Provider:** Computer			**Shared decision making:** N/A
				**Other health literacy outcome:** N/A

A number of barriers and drivers were identified that related to the providers ability to provide SNAPW health literacy interventions. The barriers and drivers can be grouped under three main headings: provider context, provider costs and interaction between providers and patients. There were 32 papers describing provider factors, 27 describing provider and service context and 20 that described barriers and drivers at the provider patient interface. The barriers and drivers are listed in Table 6. Provider barriers included lack of knowledge or skills in preventive medicine and provider attitudes to providing this type of care. Linked to this were barriers and drivers around provider context such as support for professional development and funding mechanisms for health education. Many of the drivers and barriers around the patient provider interface relate to their relationship, trust and continuity of care.

**Table 6:** Provider drivers and barriers to interventions directed at improving SNAPW and health literacy

**Barriers and Drivers (Number of Papers citing)**	**Driver**	**Barrier**
**Provider Factors€**		
Time (16)		✓
Preventive medicine skills (12)	✓	
Knowledge of guidelines / information (11)	✓	
Attitudes and beliefs (10)		✓
Teamwork / working with other providers (6)	✓	
Knowledge of referral services (3)	✓	
Confidence (2)	✓	
Communication skills (2)	✓	
Outcome expectations (2)		✓
**Provider Service Context**		
Training (duration, MI, communication), CPD, funding time, organization) (7)	✓	
Funding for health education (4)	✓	
Community links / community oriented services (2)	✓	
Availability of referral services/networks (2)	✓	✓
Information systems (2)	✓	
Availability of space (1)		✓
Supportive public health policy (1)	✓	
**Patient provider interface**		
Access of health education / lifestyle modification		
· Cost (6)		✓
· Transport (1)		✓
· Availability (3)		✓
· Physical access (1)		✓
Trust of information (6)	✓	
Continuity of care (7)	✓	
Patient provider relationship (6)	✓	
Proactive follow up (4)	✓	
Aids (e.g. Pedometer ) (4)	✓	
Reading age of materials (3)		✓
Patient education materials tailored (2)	✓	
Cultural materials / translation (2)	✓	
Individualised format (2)	✓	
Health seeking behaviour (2)		✓
Group format (1)	✓	
Goal setting (1)	✓	
Decision support (1)	✓	
Standardised assessment questions/protocols (1)	✓	

## Discussion

The results from this review highlight the complex relationships between providers and interventions to develop health literacy of patients to make SNAPW lifestyle changes. The relative effectiveness of non medical members of the primary health care medical team compared with doctors in improving health literacy was confounded by the intensity of the intervention (in terms of hours or number of contacts). Thus effectiveness in terms of improvement of health literacy may be related to capacity of the provider (time as well as skills and attitudes) to undertake more than brief interventions. For some SNAPW lifestyle changes, such as smoking cessation interventions, low intensity interventions resulted in behaviour change but not necessarily improvements in health literacy.

Shared decision making and good communication are important to developing a sense of trust and partnerships to develop health literacy [12,16] and the more intensive interventions may provide a platform for this to occur. The results from the driver and barrier extraction highlight the importance of continuity of care, the provider patient relationship and opportunity for follow up. This would support the suggestion that developing health literacy around SNAPW takes time and therefore a medium to high intensity intervention is required. Many of the barriers to shared decision making in practice, such as time, are more acute for doctors than for other health professionals [84].

Because of the nature of general practice, interventions involving doctors tend to be brief interventions and focus on issues such as smoking and physical activity prescription [19,32-35]. In Australia, initiatives such as Lifescripts (evidence based interventions to support lifestyle risk factor assessment and management), and 45+ health checks (health assessments targeting people aged 45+ at risk of developing a chronic disease) aim to support these brief interventions in primary care. Referral to programs for dietary education would provide patients at risk with a more intensive intervention but in Australia GPs only refer around 10% of their at risk patients to such programs [18,85] and GPs do not have capacity to provide more intensive interventions themselves [86]. A recent randomised controlled trial of lifestyle risk factor management in Australian general practice found that brief advice for physical activity resulted in an increase in patient self-reported activity but only those patients referred to the group programs demonstrated an improvement in diet and weight (Harris et al, MJA in press).

Creating a time where issues such as health literacy or lifestyle risk factor management can be addressed without the pressure to treat an acute problem is important and may offer an explanation as to why these more intensive approaches might be effective. Health screening programs delivered in primary health care could provide an opportunity for other members of the health care team such as practice nurses to be involved in the assessment, brief intervention, referral and group programs located at the practice. Allied health professionals such as dieticians, educators or physiotherapists could also be involved in providing education and health coaching. In addition to this there needs to be a shift in patient attitudes to using primary health care services for prevention of chronic illness. Research has shown that low health literacy is associated with poorer uptake of screening for colorectal cancer [87], breast cancer and prevention measures such as flu vaccination [88].

At a policy level there needs to be greater understanding of the skills and intensity of interventions required to improve health literacy and for SNAPW risk factor modification. For example, brief interventions can be very effective for smoking cessation [82] and this can be provided by a GP or practice nurse [51]. For more complex interventions such as dietary advice and weight loss then well trained health professionals who are able to deliver interventions of the appropriate intensity are required. Many of these interventions were group based programs which also provided peer support to the participants [36,37,39-42,44-49,52]. Educating health professionals about the impact of health literacy on a range of behaviours is important if they are to be better able to support their patients to manage their health [20,22,89]. Many of the current tools to measure health literacy may be impractical for use as a screening tool in general practice but are useful as broad guidelines to help health professionals understand the impact of low health literacy on their patient’s health status [8]. Internationally, a number of governments have policy in place to address health inequities that result from poor health literacy [9,90,91].

The main limitation of this review was that whilst there were 52 studies included, once the principal health professionals providing the intervention were identified the numbers of papers in each group were small and most of the included studies were of moderate quality, only 11/52 were assessed as being of high quality. The heterogeneity of the interventions identified also meant that a meta-analysis was not appropriate. In addition, the details of providers and description of their characteristics and role in the intervention was not systematically reported by the studies included in the review. Another limitation was the way in which health literacy is and is not measured in studies of lifestyle risk factor modification. In order to capture the complex definition of health literacy proposed by Nutbeam [5,12] then the measures need to go beyond simple measures of functional health literacy. The measures used in many of these studies included self-efficacy and patient activation in order to include those that addressed critical health literacy.

## Conclusion

The results of this review highlight the importance of the provider being able to provide moderate to high intensive interventions to address health literacy to make SNAPW lifestyle risk factor changes. As the context of the primary health care setting makes it difficult for GPs to provide the intensity of intervention required to influence health literacy and behaviour change it is important the referral mechanisms to intensive programs or other health professionals are available.

## Abbreviations

COAG, Council of Australian Governments; ABHI, Australian Better Health Initiative; SNAPW, Smoking, nutrition, alcohol, physical activity and weight; GP, general practitioner; CINAHL, Cumulative index to nursing and allied health literature; APAIS, Australian Public Affairs Information Service; TOFHLA, Test of Functional Health Literacy in Adults; REALM, Rapid Estimate of Adult Literacy in Medicine; HALS, Health Activity Literacy Scale; NVS, Newest Vital Sign.

## Competing interests

The authors declare that they have no competing interests.

## Authors’ contributions

SD, JT and AW developed and carried out the database searches. AW and JT carried out the title and abstract screen, study verification and data extraction. SD carried out the quality assessment and SD and MH extracted the drivers and barriers data. JT, AW and SD performed the statistical analysis. All authors were involved in the review conception, and participated in its design and coordination. SD wrote the manuscript and all authors read and approved the final manuscript.

## Pre-publication history

The pre-publication history for this paper can be accessed here:

http://www.biomedcentral.com/1471-2296/13/44/prepub

## Supplementary Material

Additional file 1 Effective Public Health Practice Project (EPHPP). Click here for file

Additional file 2 PRISMA 2009 checklist. Click here for file

## References

[B1] Commonwealth of Australia Building a 21st Century Primary Health Care System Australia’s First National Primary Health Care Strategy 2010 Commonwealth of Australia, Canberra

[B2] World Health Organization Preventing Chronic Disease: a Vital Investment 2007 WHO Press

[B3] Australian Institute of Health and Welfare The Burden of Disease and Injury in Australia 2000

[B4] Joint WHO/FAO Expert Consultation on Diet NatPo Diseases C Diet, Nutrition and The Prevention of Chronic Diseases: Report of a Joint Who/Fao Expert Consultation 2002, Geneva, Switzerland

[B5] Nutbeam D Kickbusch I Advancing health literacy: a global challenge for the 21st century Heal Promot Int 2000 15 3 183 184 10.1093/heapro/15.3.183

[B6] Australian Bureau of Statistics Health Literacy, Australia 2006 Australian Bureau of Statistics, Canberra

[B7] Australian Bureau of Statistics Adult Literacy and Life Skills Survey: User Guide 2006 Australian Bureau of Statistics, Canberra

[B8] Adams RJ AS Hill CL Dodd M Finlay C Wilson DH Risks associoated with low functional health literacy in an Australian Population Med J Aus 2009 191 530 534 10.5694/j.1326-5377.2009.tb03304.x 19912083

[B9] Institute of Medicine: Health Literacy Neilsen-Bohlman L, Panzer A, Kindig D A Prescription to End Confusion 2004 National Academies Press, Washington, DC 25009856

[B10] Williams J Clemens S Oleinikova K Tarvin K The Skills for Life Survey. A National Needs and Impact Survey of Literacy, Numeracy and ICT Skills 2003 Department for Education and Skills, London

[B11] Sheida White Assessing the Nation’s Health Literacy. Key Concepts and Findings of the National Assessment of Adult Literacy (NAAL) 2008 American Medical Association Foundation,

[B12] Nutbeam D Health literacy as a public health goal: A challenge for contemporary health education and communication strategies into the 21st century Heal Promot Int 2000 15 3 259 267 10.1093/heapro/15.3.259

[B13] DeWalt DA Berkman ND Sheridan S Lohr KN Pignone MP Literacy and health outcomes a systematic review of the literature J Gen Intern Med 2004 19 1228 1239 10.1111/j.1525-1497.2004.40153.x 15610334 PMC1492599

[B14] Berkman ND DA D Pignone MP Sheridan SL Lohr KN Lux L Sutton SF Swinson T Bonito AJ Literacy and Health Outcomes Evidence Report/Technology Assessment 2004 Agency for Healthcare Research and Quality, Rockville PMC4781151 15819598

[B15] Williams MV Baker DW Parker RM Nurss JR Relationship of functional health literacy to patients’ knowledge of their chronic disease. a study of patients with hypertension and diabetes Arch Intern Med 1998 158 166 172 10.1001/archinte.158.2.166 9448555

[B16] Schillinger D Bindman A Wang F Stewart A Piette J Functional health literacy and the quality of physician-patient communication among diabetes patients Patient Edu Counseling 2004 52 3 315 323 10.1016/S0738-3991(03)00107-1 14998602

[B17] National Preventative Health Taskforce Australia: The Healthiest Country by 2020 - National Preventative Health Strategy - the roadmap for action 2009 Australian Government Department of Health and Ageing, Canberra

[B18] Britt H Miller G Charles J General Practice Activity in Australia 2008–09 2009 AIHW, Canberra

[B19] Slama K Karsenty S Hirsch A French general practitioners’ attitudes and reported practices in relation to their participation and effectiveness in a minimal smoking cessation programme for patients Addiction 1999 94 1 125 132 10.1046/j.1360-0443.1999.9411259.x 10665104

[B20] Bodenheimer T Helping patients improve their health-related behaviors: what system changes do we need? Dis Manag 2005 8 5 319 330 10.1089/dis.2005.8.319 16212517

[B21] Calnan M Examining the general practitioner’s role in health education: A critical review Fam Pract 1988 5 3 217 223 10.1093/fampra/5.3.217 3066677

[B22] Crebolder HFJM Van Der Horst FG Anticipatory care and the role of Dutch general practice in health promotion - A critical reflection Patient Edu Counseling 1996 28 1 51 55 10.1016/0738-3991(96)00869-5 8852207

[B23] Dennis S May J Perkins D Zwar N Sibbald B Hasan I What evidence is there to support skill mix changes between GPs, pharmacists and practice nurses in the care of elderly people living in the community? Aust New Zealand Health Policy 2009 6 1 23 10.1186/1743-8462-6-23 19744350 PMC2749853

[B24] Zwar N Dennis S Griffiths R Perkins D May J Sibbald B Caplan G Harris M Optimising skill mix in the primary health care workforce for the care of older Australians: A systematic review APHCRI Stream 6 Reports 2007 Australian Primary Health Care Research Institute (APHCRI), Canberra

[B25] Institute for Healthcare Improvement 90-day research and development process

[B26] Prochaska JOD Carlo C Theoretical therapy: Toward a more integrative model of change Psychotherapy: Theory, Res & Pract 1982 19 3 276 288

[B27] Effective Public Health Practice Project Quality Assessment Tool For Quantitative Studies. Retrieved October 2008 from above web address. Corresponding article of interest: Thomas, B.H., Ciliska, D., Dobbins, M., & Micucci, S. (2004). A process for systematically reviewing the literature: Providing the research evidence for public health nursing interventions Worldviews on Evidence-Based Nursing 1998 1 3 176 184 10.1111/j.1524-475X.2004.04006.x 17163895

[B28] Dennis S Zwar N Griffiths R Roland M Hasan I Powell DG Harris M Chronic disease management in primary care: from evidence to policy Med JAust 2008 188 8 Suppl S53 S56 18429737 10.5694/j.1326-5377.2008.tb01745.x

[B29] Weingarten SR Henning JM Badamgarav E Knight K Hasselblad V Gano A Ofman JJ Interventions used in disease management programmes for patients with chronic illness---which ones work? Meta-analysis of published reports BMJ 2002 325 7370 925 10.1136/bmj.325.7370.925 12399340 PMC130055

[B30] Harris M Taggart J Williams A Dennis S Newall AT Shortus T Denney-Wilson E Zwar N Effective determinants for supporting lifestyle health literacy and self management skills in primary health care APHCRI Stream 13 reports 2010 Australian Primary Health Care Institute, Canberra

[B31] Liberati A Altman DG Tetzlaff J Mulrow C Gotzsche PC Ioannidis JPA Clarke M Devereaux PJ Kleijnen J Moher D The PRISMA statement for reporting systematic reviews and meta-analyses of studies that evaluate healthcare interventions: explanation and elaboration BMJ 2009 339 jul21_1 b2700 19622552 10.1136/bmj.b2700 PMC2714672

[B32] Goldstein MG Pinto BM Marcus BH Lynn H Jette AM Rakowski W McDermott S DePue JD Milan FB Dube C Physician-based physical activity counseling for middle-aged and older adults: a randomized trial Ann Behavioral Med 1999 21 1 40 47 10.1007/BF02895032 18425653

[B33] Norris SL Grothaus LC Buchner DM Pratt M Effectiveness of physician-based assessment and counseling for exercise in a staff model HMO Preventive Med 2000 30 6 513 523 10.1006/pmed.2000.0673 10901494

[B34] Beresford SASJC Kristal AR Lazovich D Feng Z Wagner EH A dietary intervention in primary care practice: the Eating Patterns Study Am J Public Health 1997 87 4 610 616 10.2105/AJPH.87.4.610 9146440 PMC1380841

[B35] Calfas K Sallis J Oldenburg B Mediators of change in physical activity following an intervention in primary care: PACE Prev Med 1997 26 297 304 10.1006/pmed.1997.0141 9144753

[B36] Adolfsson ET Walker-EngstrÃ¶m ML Smide B Wikblad K Patient education in type 2 diabetes-A randomized controlled 1-year follow-up study Diabetes ResClin Practice 2007 76 3 341 350 10.1016/j.diabres.2006.09.018 17069923

[B37] Aldana SG Greenlaw RL Diehl HA Salberg A Merrill RM Ohmine S Thomas C Effects of an intensive diet and physical activity modification program on the health risks of adults J Am Diet Assoc 2005 371 381 15746824 10.1016/j.jada.2004.12.007

[B38] Brassington GS Atienza AA Perczek RE DiLorenzo TM King AC Intervention-related cognitive versus social mediators of exercise adherence in the elderly Am J Preventive Med 2002 23 2 Suppl 80 86 10.1016/s0749-3797(02)00477-4 12133741

[B39] Dunn AL Marcus BH Kampert JB Garcia ME Kohl HW Blair SN Reduction in cardiovascular disease risk factors: 6-month results from Project Active Preventive Med 1997 883 892 10.1006/pmed.1997.0218 9388801

[B40] Efraimsson EÃ Hillervik C Ehrenberg A Effects of COPD self-care management education at a nurse-led primary health care clinic Scand J Caring Sci 2008 22 2 178 185 10.1111/j.1471-6712.2007.00510.x 18489687

[B41] Ridgeway NA Harvill DR Harvill LM Falin TM Forester GM Gose OD Improved control of type 2 diabetes mellitus: A practical education/behavior modification program in a primary care clinic Southern Medl J 1999 92 7 667 672 10.1097/00007611-199907000-00004 10414474

[B42] Sadur CN Moline N Costa M Michalik D Mendlowitz D Roller S Watson R Swain BE Selby JV Javorski WC Diabetes management in a health maintenance organization. Efficacy of care management using cluster visits Diabetes Care 1999 22 12 2011 2017 10.2337/diacare.22.12.2011 10587835

[B43] Van Sluijs EMF Van Poppel MNM Twisk JWR Van Mechelen W Physical activity measurements affected participants’ behavior in a randomized controlled trial J Clin Epidemiol 2006 59 4 404 411 10.1016/j.jclinepi.2005.08.016 16549263

[B44] Boylan MJ Renier CM Knuths JS Haller IV Preventing cardiovascular disease in women: an intervention-control randomized study Minnesota Med 2003 86 5 52 56 15495678

[B45] Miller CK Edwards L Kissling G Sanville L Evaluation of a theory-based nutrition intervention for older adults with diabetes mellitus J Am Diet Assoc 2002 102 8 1069 1081 12171451 10.1016/s0002-8223(02)90242-7

[B46] Toobert DJ Glasgow RE Strycker LA Barrera M Ritzwoller DP Weidner G Long-term effects of the Mediterranean lifestyle program: a randomized clinical trial for postmenopausal women with type 2 diabetes (Provisional abstract) Int J Behav Nutr Phys Act 2007 10.1186/1479-5868-4-1 PMC1783667 17229325

[B47] Collins R Lee RE Albright CL King AC Ready to be physically active? the effects of a course preparing low-income multiethnic women to be more physically active Health Educ Behav 2004 31 1 47 64 10.1177/1090198103255529 14768657

[B48] Winkleby MA Howard-Pitney B Albright CA Bruce B Kraemer HC Fortmann SP Predicting achievement of a low-fat diet: a nutrition intervention for adults with low literacy skills Preventive Med 1997 26 6 874 882 10.1006/pmed.1997.0231 9388800

[B49] Agurs-Collins T Kumanyika S Ten Have T Adams-Campbell L A randomised controlled trial of weight reduction and exercise for diabetes management in older African-American subjects Diabetes Care 1997 20 10 1503 1511 10.2337/diacare.20.10.1503 9314625

[B50] Delichatsios H Hunt M Lobb R EatSmart: efficacy of a multifaceted preventive nutrition intervention in clinical practice Prev Med 2001 33 91 98 11493041 10.1006/pmed.2001.0848

[B51] Lancaster T Dobbie W Vos K al e Randomized trial of nurse-assisted strategies for smoking cessation in primary care Br J Gen Pract 1999 49 191 194 10343421 PMC1313370

[B52] Siero F Broer J Bemelmans WJ Meyboom-de Jong BM Impact of group nutrition education and surplus value of Prochaska-based stage-matched information on health-related cognitions and on Mediterranean nutrition behavior Health Educ Res 2000 15 635 647 10.1093/her/15.5.635 11184222

[B53] de Vries H Kremers SP Smeets T Brug J Eijmael K The effectiveness of tailored feedback and action plans in an intervention addressing multiple health behaviors Am J Health Promotion : AJHP 2008 417 425 10.4278/ajhp.22.6.417 18677882

[B54] Hartman TJ McCarthy PR Park RJ Schuster E Kushi LH Results of a community-based low-literacy nutrition education program J Community Health 1997 22 5 325 341 10.1023/A:1025123519974 9353681

[B55] Kloek GC van Lenthe FJ van Nierop PWM Koelen MA Mackenbach JP Impact evaluation of a Dutch community intervention to improve health-related behaviour in deprived neighbourhoods Health and Place 2006 12 4 665 677 10.1016/j.healthplace.2005.09.002 16253541

[B56] Lorig KR Ritter PL Jacquez A Outcomes of border health Spanish/English Chronic Disease Self-management Programs Diabetes Educator 2005 31 3 401 409 10.1177/0145721705276574 15919640

[B57] Nies MA Artinian NT Schim SM Vander Wal JS Sherrick-Escamilla S Effects of lay health educator interventions on activity, diet, and health risks in an urban Mexican American community J Prim Prev 2004 25 4 441 455

[B58] Yajima S Takano T Nakamura K Watanabe M Effectiveness of a community leaders' programme to promote healthy lifestyles in Tokyo, Japan Heal Promot Int 2001 16 3 235 243 10.1093/heapro/16.3.235 11509459

[B59] Jimmy G Martin BW Implementation and effectiveness of a primary care based physical activity counselling scheme Patient Edu Counseling 2005 56 3 323 331 10.1016/j.pec.2004.03.006 15721975

[B60] Kreuter MW Strecher VJ Do tailored behavior change messages enhance the effectiveness of health risk appraisal? Results from a randomized trial Heal Educ Res 1996 11 1 97 105 10.1093/her/11.1.97 10160231

[B61] Oenema A Brug J Dijkstra A de Weerdt I de Vries H Efficacy and use of an internet-delivered computer-tailored lifestyle intervention, targeting saturated fat intake, physical activity and smoking cessation: a randomized controlled trial Annals of Behavioral Medicine : a Publication of the Society of Behavioral Medicine 2008 125 135 18363076 10.1007/s12160-008-9023-1

[B62] Ogden J Hoppe R The relative effectiveness of two styles of educational package to change practice nurses' management of obesity Int J Obes 1997 21 11 963 971 10.1038/sj.ijo.0800503 9368818

[B63] Simmons D Voyle JA Fout F Feot S Leakehe L Tale of two churches: Differential impact of a church-based diabetes control programme among Pacific Islands people in New Zealand Diabet Med 2004 21 2 122 128 10.1111/j.1464-5491.2004.01020.x 14984446

[B64] Smith C Ryan A Change for Life/Cambia tu vida: a health promotion program based on the stages of change model for African descendent and Latino adults in New Hampshire Preventing Chronic Disease 2006 3 3 1 11 PMC1637793 16776866

[B65] Steptoe A Rink E Kerry S Psychosocial predictors of changes in physical activity in overweight sedentary adults following counseling in primary care Preventive Med 2000 31 2 I 183 194 10.1006/pmed.2000.0688 10938220

[B66] Hoffman AM Redding CA Goldberg D Añel D Prochaska JO Meyer PM Pandey D Computer expert systems for African-American smokers in physicians offices: A feasibility study Preventive Med 2006 43 3 204 211 10.1016/j.ypmed.2006.03.025 16780939

[B67] Wolf RL Lepore SJ Vandergrift JL Basch CE Yaroch AL Tailored telephone education to promote awareness and adoption of fruit and vegetable recommendations among urban and mostly immigrant black men: A randomized controlled trial Preventive Med 2009 48 1 32 38 10.1016/j.ypmed.2008.10.015 PMC4537646 19010349

[B68] Swerissen H Belfrage J Weeks A Jordan L Walker C Furler J McAvoy B Carter M Peterson C A randomised control trial of a self-management program for people with a chronic illness from Vietnamese, Chinese, Italian and Greek backgrounds Patient Edu Counseling 2006 64 1–3 360 368 10.1016/j.pec.2006.04.003 16859871

[B69] Campbell MK DeVellis BM Strecher VJ Ammerman AS DeVellis RF Sandler RS Improving dietary behavior: the effectiveness of tailored messages in primary care settings Am J Public Health 1994 84 5 783 787 10.2105/AJPH.84.5.783 8179049 PMC1615043

[B70] Block G Wakimoto P Mandel R Metz D Fujii ML Feldman N Sutherland B A randomized trial of the little by little cd-rom: demonstrated effectiveness in increasing fruit and vegetable intake in a low-income population Preventing Chronic Disease 2004 1 3 1 12 PMC1253473 15670429

[B71] Koffman DMBT Mosca L Redberg R Schmid T Wattigney WA An evaluation of Choose to Move 1999: an American Heart Association physical activity program for women Arch Intern Med 2001 161 18 2193 2199 10.1001/archinte.161.18.2193 11575975

[B72] Marcus BHBB Pinto BM Roberts LH Forsyth MB Traficante RM Efficacy of an individualized, motivationally-tailored physical activity intervention Ann Behav Med 1998 20 3 174 180 10.1007/BF02884958 9989324

[B73] Prochaska JO Velicer WF Redding C Rossi JS Goldstein M DePue J Greene GW Rossi SR Sun X Fava JL Stage-based expert systems to guide a population of primary care patients to quit smoking, eat healthier, prevent skin cancer, and receive regular mammograms Preventive Med 2005 41 2 406 416 10.1016/j.ypmed.2004.09.050 15896835

[B74] Prochaska JO Velicer WF Rossi JS Redding CA Greene GW Rossi SR Sun X Fava JL Laforge R Plummer BA Multiple risk expert systems interventions: impact of simultaneous stage-matched expert system interventions for smoking, high-fat diet, and sun exposure in a population of parents Heal Psychol 2004 23 5 503 516 10.1037/0278-6133.23.5.503 15367070

[B75] Marcus BHEKM Simkin-Silverman LR Linnan LA Taylor ER Bock BC Roberts MB Rossi JS Abrams DB Evaluation of motivationally tailored vs. standard self-help physical activity interventions at the workplace Am J Heal Promot 1998 12 4 246 253 10.4278/0890-1171-12.4.246 10178617

[B76] Graham-Clarke P Oldenburg B The effectiveness of a general-practice-based physical activity intervention on patient physical activity status Behav Chang 1994 11 3 132 144

[B77] O'Loughlin J Paradis G Meshefedjian G Kishchuk N Evaluation of an 8-Week mailed healthy-weight intervention Preventive Med 1998 27 2 288 295 10.1006/pmed.1998.0265 9579009

[B78] Butler C Rollnick S Cohen D Motivational consulting versus brief advice for smokers in general practice: a randomized trial Br J Gen Pract 1999 49 611 616

[B79] Fries E Edinboro P McClish D Manion L Bowen D Beresford S Ripley J Randomized trial of a low-intensity dietary intervention in rural residents: the Rural Physician Cancer Prevention Project Am J Prev Med 2005 28 162 168 10.1016/j.amepre.2004.10.017 15710271

[B80] Little P Dorward M Gralton S Hammerton L Pillinger J White P A randomised controlled trial of three pragmatic approaches to initiate increased physical activity in sedentary patients with risk factors for cardiovascular disease Br J Gen Pract 2004 54 500 189 195 15006124 PMC1314829

[B81] Naylor P Simmonds G Riddoch C Comparison of stage-matched and unmatched interventions to promote exercise behaviour in the primary care setting Health Educ Res 1999 14 653 666 10.1093/her/14.5.653 10510073

[B82] Slama K Redman S Perkins J al E The effectiveness of two smoking cessation programmes for use in general practice: a randomised controlled trial BMJ 1999 300 1707 1709 2202445 10.1136/bmj.300.6741.1707 PMC1663307

[B83] Lennox AS Osman LM Reiter E Robertson R Friend J McCann I Skatun D Donnan PT Cost effectiveness of computer tailored and non-tailored smoking cessation letters in general practice: randomised controlled BMJ 2001 322 7299 1396 10.1136/bmj.322.7299.1396 11397745 PMC32255

[B84] Légaré F Ratté S Gravel K G ID Barriers and facilitators to implementing shared decision-making in clinical practice: Update of a systematic review of health professionals perceptions Patient Edu Counseling 2008 73 3 526 535 10.1016/j.pec.2008.07.018 18752915

[B85] Amoroso C Harris M Ampt M Laws R McKenzie S Williams A Jayasinghe U Zwar N Powell Davies G Health check for 45–49 year old patients in general practice: feasibility and impact on practices and patient behaviour Aust Family Physician 2009 38 358 362 19458808

[B86] Harris M Hobbs C Powell Davies G Implementation of a SNAP intervention in two divisions of general practice: a feasibility study Med J Aust 2005 183 s54 s58 16296953 10.5694/j.1326-5377.2005.tb07180.x

[B87] Miller David P Brownlee Caroline D McCoy Thomas P Pignone MP The effect of health literacy on knowledge and receipt of colorectal cancer screening: a survey study BMC Fam Pract 2007 8 16 10.1186/1471-2296-8-16 17394668 PMC1852801

[B88] Berkman ND Sheridan SL Donahue KE Halpern DJ Crotty K Low health literacy and health outcomes: an updated systematic review Ann Intern Med 2011 155 2 97 107 21768583 10.7326/0003-4819-155-2-201107190-00005

[B89] Bowler I Gooding S Health promotion in primary health care: The situation in England Patient Edu Counseling 1995 25 3 293 299 10.1016/0738-3991(95)00803-8 7630833

[B90] Rootman I Gordon-El-Bihbety D A Vision for a Health Literate Canada Report of the Expert Panel on Health Literacy 2008 Canadian Public Health Association, Ottawa

[B91] Parker RM Ratzan SC Lurie N Health literacy: a policy challenge for advancing high-quality health care Health Aff 2003 22 4 147 153 10.1377/hlthaff.22.4.147 12889762

